# Improvement of peripheral neuropathy in a patient with antineutrophil cytoplasmic antibody-negative eosinophilic granulomatosis with polyangiitis by additional mepolizumab

**DOI:** 10.1186/s13223-022-00653-7

**Published:** 2022-02-19

**Authors:** Yoshiro Kai, Masanori Yoshikawa, Masayuki Matsuda, Kentaro Suzuki, Hiroya Ohara, Naohiko Iguchi, Takehito Kasamatsu, Kenji Uno, Nobuhiro Fujioka, Yukio Fujita, Shigeo Muro

**Affiliations:** 1 Department of Respiratory Medicine, Minami-Nara General Medical Center, 8-1 Fukugami, Oyodo-cho, Yoshino-gun, Nara, 638-8551 Japan; 2 Department of Respiratory Medicine, Nara Medical University, 840 Shijo-cho, Kashihara City, Nara, 634-8522 Japan; 3 Department of Neurology, Minami-Nara General Medical Center, 8-1 Fukugami, Oyodo-cho, Yoshino-gun, Nara, 638-8551 Japan; 4 Department of Neurology, Nara Medical University, 840 Shijo-cho, Kashihara City, Nara, 634-8522 Japan; 5 Department of Infectious Diseases, Minami-Nara General Medical Center, 8-1 Fukugami, Oyodo-cho, Yoshino-gun, Nara, 638-8551 Japan

**Keywords:** Asthma, Eosinophilic granulomatosis with polyangiitis, IL-5, Mepolizumab, Peripheral neuropathy

## Abstract

**Background:**

Eosinophilic granulomatosis with polyangiitis (EGPA) is a vasculitis characterized by abnormally high eosinophils and frequent peripheral neuropathy. Mepolizumab is an approved therapy for EGPA, but its efficacy against peripheral neuropathy remains unknown.

**Case presentation:**

A 41-year-old woman was admitted in the hospital with dyspnea and neuropathy. Ground glass opacity and infiltrative shadow in the bilateral lungs were evident on chest computed tomography images. Eosinophils were increased in serum, in bronchoalveolar lavage fluid (BALF), and in transbronchial lung biopsy, and bacteria were not detected in BALF. EGPA resulting in severe eosinophilic asthma, sinusitis, pulmonary infiltrates, and peripheral neuropathy was diagnosed. Prednisolone (50 mg/day) caused remission of eosinophilic pneumonia and sinusitis, but not peripheral neuropathy. During prednisolone tapering (7 mg/day, 10 months after treatment), eosinophils were increased, and peripheral neuropathy relapsed. The humanized anti-IL-5 antibody mepolizumab (300 mg) was initially administered, followed by prednisolone. Mepolizumab caused sustained peripheral neuropathy remission and effective prednisolone tapering.

**Conclusions:**

Introduction of mepolizumab combined with prednisolone may improve peripheral neuropathy.

## Background

Eosinophilic granulomatosis with polyangiitis (EGPA) is a type of antineutrophil cytoplasmic antibody (ANCA)-associated vasculitis. It is characterized by severe asthma and eosinophilia of the blood and tissue. The usual treatment involves prednisolone and immunosuppressants such as cyclophosphamide. However, relapses are common in prednisolone tapering.

Peripheral neuropathy is often observed in patients with EGPA and is significantly associated with poor quality of life [1, 2]. An anti-interleukin (IL)-5 monoclonal antibody, mepolizumab, has been approved for the treatment of EGPA. IL-5 is a cytokine that is essential for eosinophil maturation and activation. The MIRRA study [3] demonstrated that mepolizumab is an effective and safe add-on therapy in participants with relapsing or refractory EGPA, allowing reductions in prednisolone dose. However, there have been few reports of the effect of mepolizumab on peripheral neuropathy. We herein describe a case of EGPA with neuropathy successfully treated with mepolizumab in addition to prednisolone.

## Case presentation

**Fig. 1:**
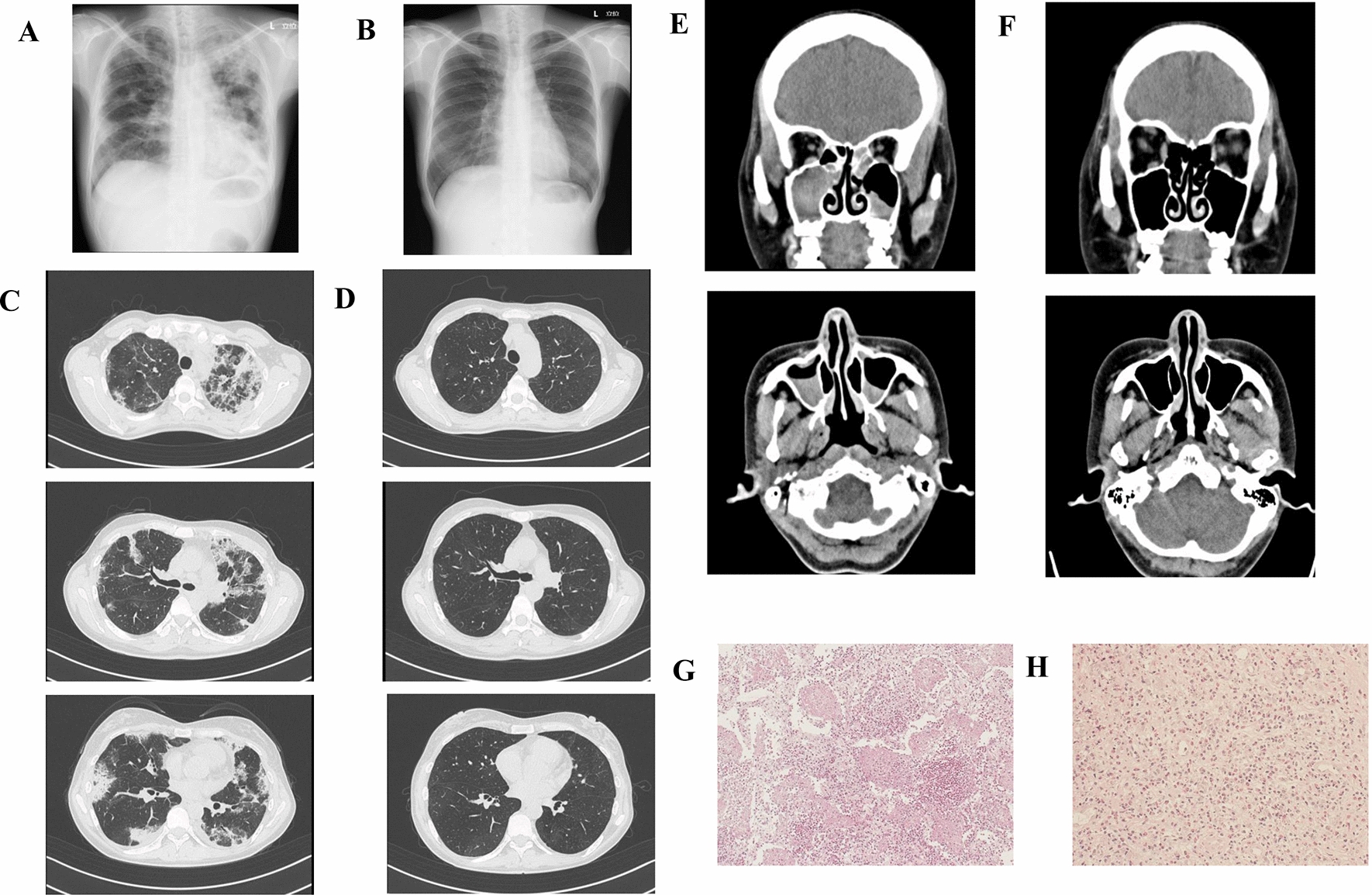
Chest X-ray, computed tomography (CT), and histology on admission and after treatment. Chest X-ray reveals (**A**) bilateral reticular shadows at admission and **B** a significant improvement three weeks after treatment. Chest CT shows (**C**) non-segmental bilateral consolidation with peripheral distribution at admission and **D** significant improvement after treatment. **E** Paranasal CT reveals dominant ethmoid sinus shadows on admission. (F) Six months after treatment, CT shows a significant improvement. Biopsies from lung (**G**) and sinus (**H**) with hematoxylin and eosin staining show eosinophilic infiltration at admission

A 41-year-old woman was diagnosed with asthma and allergic rhinitis five years ago. She received medium-dose inhaled corticosteroids, long-acting β2-agonists, and leukotriene receptor antagonists. Her asthma gradually deteriorated. During the last month before her admission in the hospital, she experienced exertional dyspnea, continuous nasal congestion, and loss of smell. During the last seven days before her admission in the hospital, she experienced dyspnea at rest and paresthesia as well as pain in the bilateral lower legs and arthralgia in both knees. She was admitted to our hospital with complaints of severe dyspnea. During admission, a wheeze could be confirmed in the bilateral lung field and the patient experienced progressive paresthesia and pain in the bilateral lower legs and arthralgia in both knees. These neurologic symptoms occurred simultaneously, while no other symptoms were observed. Neurological examination revealed normal cranial nerve and manual muscle testing results were normal. The patient never experienced any neuropathic or residual neuropathic symptoms. Her blood pressure was 94/62 mmHg, pulse rate was 109 beats/minute, respiratory rate was 20 breaths/minute, oxygen saturation was 95% on room air, and body temperature was 36.7 °C. Laboratory findings revealed a white blood cell count of 22,200/μL with 66.7% eosinophils, hemoglobin 10.8 g/dL, C-reactive protein 8.48 mg/dL, and IgE 722 IU/mL. Myeloperoxidase ANCA and proteinase-3 (PR3)-ANCA were within normal ranges. Arterial blood examination at room air showed a pH of 7.434, partial pressure of carbon dioxide (PaCO2) of 38.8 mmHg, partial pressure of oxygen (PaO2) of 73.5 mmHg, and bicarbonate (HCO3−) of 25.9 mEq/L. A chest X-ray revealed reticular shadows in the bilateral lungs (Fig. 1A), which improved after the initial prednisolone treatment (Fig. 1B). Computed tomography revealed ground glass opacity and consolidation in the bilateral lung fields (Fig. 1C) and paranasal sinusitis (Fig. 1E), which improved after prednisolone treatment (Fig. 1D, F). Bronchoalveolar lavage fluid (BALF) contained a high percentage of eosinophils (90.0%). Transbranchial lung biopsy specimens showed eosinophilic infiltration (Fig. 1G); the same was also observed on nasal polyp biopsy (Fig. 1H). Electrocardiogram and echocardiography were normal. Magnetic resonance imaging revealed no dural thickening related to hypertrophic pachymeningitis. The patient was diagnosed with EGPA as her condition matched the characteristic clinical course described by the American College of Rheumatology classification criteria [4] with symptoms due to vasculitis following asthma and elevated eosinophils in the peripheral blood.

**Fig. 2:**
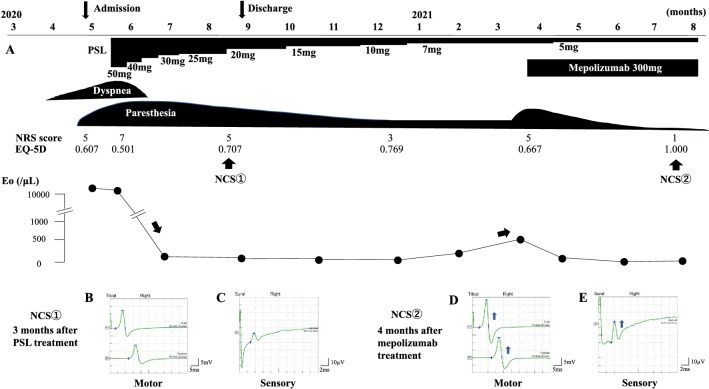
Clinical course of the case study and motor and sensory nerve conduction study. **A** With prednisolone (PSL) treatment, clinical symptoms and peripheral blood eosinophil count decreased. Ten months after administration of prednisolone, signs of relapse were observed and mepolizumab was added. Nerve conduction study (NCS) was performed 3 months after prednisolone (PSL) treatment (NCS①) and 4 months after mepolizumab treatment (NCS②). Motor nerve conduction amplitude in the right tibial nerves (ankle and popliteal) was reduced 3 months after prednisolone treatment (**B**), and improved 4 months after mepolizumab treatment (**D**). Similarly, sensory nerve conduction amplitude in the right sural nerves (ankle and popliteal) was decreased 3 months after prednisolone treatment (**C**) and improved 4 months after mepolizumab treatment (**E**). PSL: prednisolone, NRS: numeric rating scale, NCS: nerve conduction study, Eo: eosinophil count

Figure 2A shows the patient’s clinical course. Pain scores on Numerical Rating Scale (NRS) were assessed [5]. Quality of life was assessed using the Euro quality of life 5-dimension (EQ-5D) -3L scale [6]. The Japanese version of EQ-5D has been validated previously [6]. On admission, NRS score was 5 and EQ-5D score was 0.607. After admission, NRS peaked to 7 and EQ-5D was minimal 0.501. The patient was initially administered with 50 mg/day of prednisolone that resulted in the improvement of symptoms. However, considerable bilateral paresthesia remained (NRS: 3, EQ-5D: 0.769), and the patient was advised for a follow-up. After prednisolone was tapered, the patient was discharged. When prednisolone was tapered to 7 mg (10 months from treatment initiation), she again experienced increased eosinophil count in the peripheral blood and recurrence of symptoms (NRS: 5, EQ-5D: 0.667) such as paresthesia and pain in the bilateral lower legs and arthralgia in both knees. She then initiated treatment with mepolizumab (300 mg every four weeks). After administration of mepolizumab, her paresthesia, pain, and arthralgia gradually improved (NRS: 1, EQ-5D: 1.000). A nerve conduction test performed 3 months after prednisolone treatment showed motor and sensory nerve conduction amplitudes were decreased to 12.7 mV, 9.2 mV, and 12.6 μV in the right tibial ankle, right tibial popliteal, and right sural nerves, respectively (Figs. 2B, C). The test was repeated after 4 months of mepolizumab treatment, at which time these conduction amplitudes increased to 18.8 mV, 13.8 mV, and 28.3 μV, respectively (Figs. 2D, E).

## Discussion and conclusions

EGPA is characterized by asthma, elevated eosinophils, and small vessel vasculitis with necrotizing granuloma [1]. In EGPA, peripheral neuropathy often develops (51.4%), usually mononeuritis multiplex [1] Peripheral neuropathy alone does not affect a patient’s survival, but is significantly related to physical function and quality of life [2]. Upon prednisolone administration, the main treatment for EGPA, most patients immediately improve, while others present refractory disease [2]. Recently, intravenous immunoglobulin (IVIG) use for treating neuropathy in EGPA refractory to prednisolone treatment has been reported [7]. However, its effects on peripheral neuropathy have not yet been established.

The 1996 Five-Factor Score for systemic necrotizing vasculitides can assess prognosis during diagnosis, using age (> 65 years), cardiac symptoms, gastrointestinal involvement, renal insufficiency, and ear, nose, and throat (ENT) symptoms [8]. In this case, the patient only presented nose symptoms, and ENT symptoms are associated with a lower relative risk of death. She was therefore initially treated only with prednisolone and not with an immunosuppressant agent such as cyclophosphamide. Prednisolone improved eosinophilic pneumonia and eosinophilic rhinosinusitis remarkably. However, it did not improve patient’s peripheral neuropathy. After administration of prednisolone, steroid-diabetes mellitus could not be detected.

During steroid tapering (7 mg prednisolone), 10 months after treatment was initiated, peripheral eosinophils started increasing, peripheral neuropathy progressed, and the patient’s quality of life deteriorated. Mepolizumab treatment was thus initiated. Neurological symptoms gradually improved as confirmed by a nerve conduction test.

Differential clinicopathologic features of EGPA-associated neuropathy with or without ANCA have recently been reported [9], with nerve biopsy specimens in the ANCA-positive group showing vasculitis resulting in ischemia and inflammation and the ANCA-negative group showing eosinophil-associated vascular occlusion leading to ischemia and eosinophil-associated tissue damage. Control of vascular eosinophil infiltration may thus be crucial.

In the MIRRA study, mepolizumab was effective for the treatment of EGPA. A large number of patients with EGPA with peripheral neuropathy were included. In this case, however, the efficacy of mepolizumab for peripheral neuropathy was not evaluated because the patient declined invasive examination of sural nerve biopsy. She did not present any comorbidities that could have contributed to neuropathy. Recently, low-dose mepolizumab has been reported to be effective as an add-on therapy for treating long-lasting peripheral neuropathy in patients with EGPA [10]. In this case, administration of mepolizumab for 9 months resulted in the absence of neuropathy recurrence.

In conclusion, mepolizumab in combination with prednisolone may effectively treat peripheral neuropathy associated with EGPA and prevent the establishment of fixed peripheral neuropathy. Further studies are required to clarify the therapeutic efficacy of mepolizumab in peripheral neuropathy associated with EGPA to establish the optimal timing for its administration.

## Data Availability

The datasets used and/or analyzed during the current study are available from the corresponding author on any reasonable request.

## Abbreviations

ANCA: Antineutrophil cytoplasmic antibody
BALF: Bronchoalveolar lavage fluid
CT: Computed tomography
EGPA: Eosinophilic granulomatosis with polyangiitis
ENT: Ear, nose, and throat
HCO3−: Bicarbonate
ICS: Inhaled corticosteroids
IL: Interleukin
IVIG: Intravenous immunoglobulin
LABA: Long-acting β2-agonists
LTRA: Leukotriene receptor antagonists
PaCO2: Partial pressure of carbon dioxide
PaO2: Partial pressure of oxygen
PR3: Proteinase-3
PSL: Prednisolone
